# Transcriptomic comparison of avian auditory and vestibular sensory epithelia

**DOI:** 10.1016/j.isci.2025.113780

**Published:** 2025-10-15

**Authors:** Mitsuo Paul Sato, Ishwar Vithal Hosamani, Stefan Heller, Marie Kubota

**Affiliations:** 1 Department of Otolaryngology – Head and Neck Surgery, Stanford University School of Medicine, Stanford, CA, USA; 2 Institute for Stem Cell Biology and Regenerative Medicine, Stanford University School of Medicine, Stanford, CA, USA; 3 Department of Otolaryngology-Head and Neck Surgery, Kindai University School of Medicine, Osaka, Japan

**Keywords:** biotechnology, molecular biology, neuroscience

## Abstract

Our inner ears contain various hair cell subtypes with distinct cytomorphologies, innervation, and functions. Here, we computationally compared hair cell transcriptomes from the avian hearing organ, the basilar papilla, and the utricle, a vestibular organ, to explore how these subtypes differ in gene expression within a single species. We identified distinct gene expression patterns in auditory and vestibular hair cell subgroups. Next, we integrated existing transcriptomic datasets from regenerated nascent auditory hair cells and nascent utricle hair cells arising during natural turnover. We found that while nascent hair cells possess unique transcriptomic profiles, they are more similar to utricular type II hair cells than to their mature functional counterparts. Additionally, three weeks after aminoglycoside-induced hair cell loss in the utricle, the regenerated hair cells lacked type I hair cell gene expression properties. This study provides fundamental insights into avian hair cell diversity and offers a basis for cross-species comparative studies.

## Highlights

• Distinct transcriptomes of auditory and vestibular hair cells in chickens
• Regenerated basilar papilla hair cells resemble type II utricle hair cells
• Nascent utricle hair cells from turnover resemble regenerated basilar papilla hair cells
• Evolutionary insight into type I and type II utricle hair cell identities

## Graphical abstract

**Figure:**
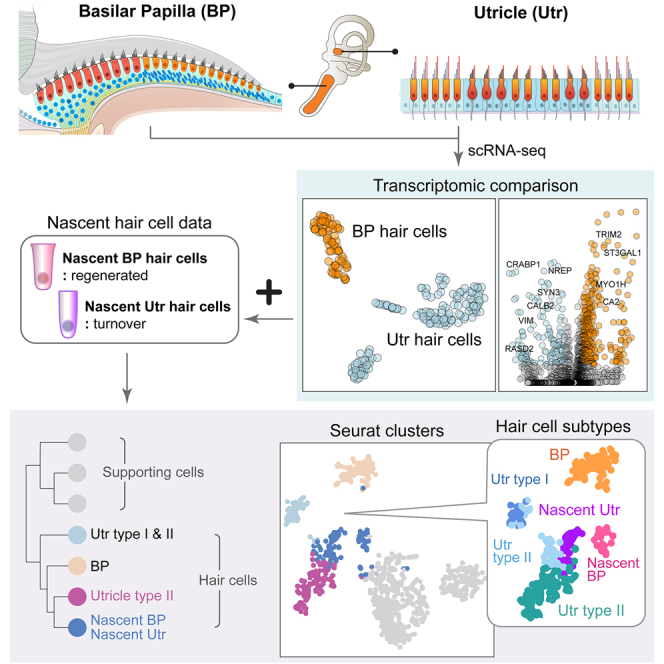

## Introduction

The senses of hearing and balance critically depend on the function of mechanosensitive hair cells. These hair cells are situated in our inner ears at locations where they can effectively detect linear acceleration (in the utricle, saccule, and lagena^1^), angular movement (in the cristae associated with the three semicircular canals), and auditory stimulation (in the cochlea). A variety of hair cell subtypes exist, differing in form and function, yet they maintain the basic apicobasal polarity and cytoarchitecture of a compact and cylindrical primary sensory neuron. The hallmark of all hair cells is their mechanosensitive hair bundle protruding from the apical surface. Additionally, hair cells possess diverse basolateral synaptic specializations linked to functionally distinct afferent and efferent innervation. Typically, hair cells are interspersed with supporting cells, which arise from a common progenitor during embryonic development.^2^

The mammalian cochlear organ of Corti contains a single row of inner hair cells and three rows of outer hair cells. The inner hair cells convert mechanical auditory stimuli into electrical signals and form synapses with afferent auditory neurons, which relay the electrical signals to the brain. The outer hair cells have much less afferent innervation and are primarily innervated by efferent nerve fibers. While also acting as signal transducers, outer hair cells amplify and modulate sound stimuli.^3^^,^^4^ Various types of supporting cells surround the inner and outer hair cells.^5^ The avian auditory organ, known as the basilar papilla, is located in the tubular cochlear duct and harbors the tall and the short hair cells, which are proposed as functional equivalents to the inner and outer hair cells of the mammalian cochlea.^6^^,^^7^^,^^8^ Tall hair cells are innervated by afferent nerves and a small proportion of efferent nerves, while short hair cells predominantly receive efferent innervation.^9^ Interestingly, a similar organization is observed in monotremes, which have a basilar papilla-like cochlear organization containing multiple rows of inner and outer hair cells, indicating an evolutionary intermediate stage between the avian and therian mammalian auditory organs.^10^

The overall morphological structure of mammalian and avian vestibules shares more similarities than differences. The utricle is the most well-studied vestibular organ. It contains two main hair cell subtypes: flask-shaped type I and cylindrical type II hair cells, which differ in their afferent synaptic connections. Type I hair cells are innervated by calyx-like synapses surrounding their basolateral surface, whereas bouton-type synaptic structures innervate type II utricle hair cells.^11^^,^^12^ Mammalian and avian utricles harbor both hair cell subtypes, with differences in their regional distribution. In birds, type I hair cells are restricted to the striolar region, whereas in mammals, they are distributed throughout the utricular sensory epithelium.^13^

Hair cells are vulnerable to damage from various stimuli, including noise exposure, certain drugs, and aging. The resulting loss of cochlear hair cells leads to irreversible hearing loss in mammals due to their lack of regenerative capacity.^14^ In contrast, non-mammalian vertebrates, such as chickens, show a strong regenerative capability. Following the demise of hair cells, the underlying supporting cells either enter the S-phase and divide to produce new hair cells or directly convert into hair cells via transdifferentiation.^15^^,^^16^ Interestingly, in addition to robust regeneration in response to hair cell damage, the avian vestibular organs display ongoing turnover of hair cells.^12^ In contrast, mammalian vestibular organs exhibit only rudimentary regenerative potential.^17^^,^^18^

The various types of hair cells evolved in response to the diverse evolutionary pressures faced by organisms inhabiting a wide range of environmental niches. Their mechanically stimulated sensory systems are adapted to a multitude of stimuli associated with changes in pressure in both air and liquids, while also developing mechanisms to measure gravitational forces and acceleration. The varied utilization of hair cells enabled life on Earth to thrive in the depths of the oceans, on land, and in the air. The refined senses of hearing and balance empower animals to avoid conflict, hunt, and communicate, among other significant achievements made possible by the use of different hair cell subtypes.

Our study focuses on two avian inner ear organs: the basilar papilla and the utricle. We concentrate on the various hair cell subtypes found in these organs. Our goal is to identify distinct marker genes and transcriptional profiles associated with the physiological functions of these hair cell subtypes. Our comparative analysis revealed distinct transcriptomic profiles for hair cells in the basilar papilla and utricle. Moreover, a comparison with newly regenerated avian auditory hair cells revealed transcriptomic characteristics similar to those of utricle type II hair cells, rather than their homeostatic counterparts, implying that these regenerated hair cells initially adopt a distinct nascent state.

We expect that our transcriptomic atlas will establish a baseline for comparison with mammalian inner ear hair cells and help understand why hair cell subtypes vary in their vulnerability to mechanical overexposure or ototoxic drugs within and across species. Cataloging these differences may also provide insights into the evolutionary branch points of these inner ear end organs, given that specific gene expression patterns are known to influence balance-sound specification during inner ear development in mammals.^19^ Furthermore, our data pave the way for detailed comparisons with other cell groups, particularly supporting cells, as regenerative potential is intricately connected to the characteristics of supporting cells.

## Results

### Chicken basilar papilla and utricle hair cells have distinctive transcriptomic profiles

**Figure 1:**
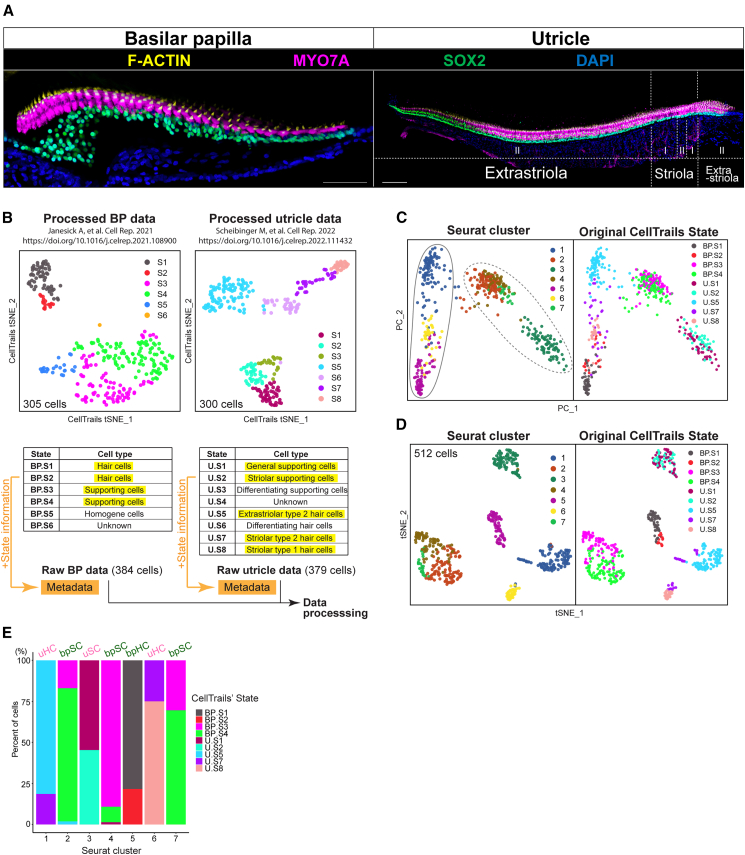
Combined single-cell RNA-seq data from chicken basilar papilla and utricle (A) Transverse sections of chicken basilar papilla (left) and utricle (right), imaged to show their anatomy, labeled with hair cell and supporting cell markers (MYO7A and SOX2, respectively), and hair bundles labeled with F-actin. Scale bar: 50 μm (left) and 100 μm (right). I: type I hair cells. II: type II hair cells. (B) T-distributed stochastic neighbor embedding (tSNE) plots showing the basilar papilla (BP) (left plot) and utricle (U) (right plot) cells, taken from our published datasets,^7^^,^^20^ visualized by CellTrails’ clusters (States). The charts show the cell types of BP and utricle corresponding to the individual States. State information of the BP and utricle datasets was added to the metadata of the individual BP and utricle raw (unprocessed) data. Only cells associated with matured hair cell- and supporting cell-States (BP.S1, BP.S2, BP.S3, BP.S4, Utricle (U).S1, U.S2, U.S5, U.S7, and U.S8: highlighted in yellow) were subsetted from these raw data (in other words, cells associated with other States or cells without State information were removed) for further analysis. (C) Principal component analysis (PCA) plot of the merged data shows the separation of hair cell clusters and supporting cell clusters, visualized by Seurat clusters (left) and the original CellTrails’ States (right). Solid and dotted ellipses indicate hair cell and supporting cell clusters, respectively. (D) T-SNE visualization of the clustering analysis of the subsetted data (512 cells) as illustrated in (B). The left panel shows 7 clusters predicted by Seurat, and the right panel shows the original CellTrails’ States mapped on the same t-SNE. (E) Bar plot shows the proportion of cells from each CellTrails’ States in individual Seurat clusters. uHC: utricle hair cells; bpSC: basilar papilla supporting cells; uSC: utricle supporting cells; bpHC: basilar papilla hair cells.

Previously published single-cell RNA-seq studies of the chicken basilar papilla and utricle have defined the transcriptomic identities of the major cell types, namely the hair cells and supporting cells, that comprise the sensory epithelia of these sensory organs (Figure 1A).^7^^,^^20^ These studies identified and validated markers for two subtypes of hair cells and two subtypes of supporting cells, along with an adjacent epithelial cell type known as homogene cells, in the basilar papilla.^7^ Similarly, three subtypes of supporting cells and four subtypes of hair cells were characterized in the avian utricle.^20^ To compare the transcriptomic landscape of the principal cell types in the basilar papilla and the utricle, we merged and analyzed the previously published datasets using the Seurat R package^21^ (Figure 1B). We excluded the homogene cell cluster and included the previously validated clusters for basilar papilla hair cells and supporting cells in our analysis. Likewise, for the utricle, we included three hair cell groups (extrastriolar type II, striolar type I, and striolar type II) and two supporting cell groups in subsequent analyses, omitting the supporting cells and hair cells that were linked to new hair cell production during natural turnover (Figure 1B).

The merged gene expression count matrices, excluding previously assigned annotations, were normalized using SCTransform in Seurat. This workflow employs a regularized negative binomial regression model to account for sequencing depth and gene-specific variation, thereby stabilizing variance across the gene expression range and making lowly expressed genes more comparable to highly expressed genes.^21^ Dimensionality reduction via principal component analysis (PCA) revealed that the first principal component robustly segregated hair cells from supporting cells, independent of their tissue of origin (Figure 1C). This separation in PCA space was validated by mapping the cells with annotation metadata imported from the published CellTrails analyses.^7^^,^^20^^,^^22^ We then performed unsupervised clustering in a reduced-dimensional space of the first ten principal components, which revealed seven distinct clusters (Figure 1D; Table S1). Within hair cell and supporting cell populations, we found that the basilar papilla and the utricle–derived cells formed distinct clusters, indicating that cells from these tissues exhibit origin-specific, unique transcriptomic profiles (Figure 1E). In the hair cell population, cluster 5 was exclusively comprised of basilar papilla hair cells (BP.S1 and BP.S2), while clusters 1 and 6 consisted of utricle hair cells (U.S5, U.S7, and U.S8). Similarly, in the supporting cell population, clusters 2, 4, and 7 included basilar papilla supporting cells (BP.S3 and BP.S4), while cluster 3 exclusively contained supporting cells from the utricle (U.S1 and U.S2) (Figures 1B–1D).

**Figure 2:**
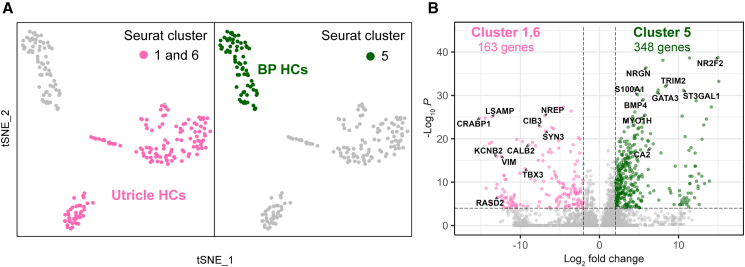
Differentially expressed genes in the basilar papilla and utricle hair cells (A) tSNE plots highlighting basilar papilla hair cells (cluster 5) and utricle hair cells (clusters 1 and 6). (B) Volcano plot showing differentially expressed (DE) genes between basilar papilla hair cells and utricle hair cells. 348 and 163 DE genes were identified in basilar papilla and utricle hair cells, respectively (Log_2_ fold change >|2| and p_val_adj <1E-4).

To identify genes that are differentially expressed in basilar papilla hair cells and utricle hair cells, we compared cluster 5 (basilar papilla) with clusters 1 and 6 (utricle) (Figure 2A). We identified 348 genes enriched in basilar papilla hair cells and 163 genes in utricle hair cells (Figure 2B; Table S2). Differential gene expression analysis between the supporting cells of the basilar papilla and those of the utricle also revealed unique gene expression profiles between these two cell groups, with 186 enriched genes in cluster 3 and 55 enriched genes in clusters 2, 4, and 7 compared to the corresponding hair cell groups (Figures S1A and S1B; Table S3).

In basilar papilla hair cells (cluster 5), “sialyltransferase activity” was the only GO term significantly enriched (adjusted *p*-value <0.05) (Table S4), indicating that the sialylation of glycan chains is significantly more prevalent in auditory hair cells in the basilar papilla compared to the vestibular hair cells of the utricle. In contrast, utricle hair cells (clusters 1, 6) displayed ten significantly enriched GO terms (adjusted *p*-value ≤0.002), which pertained exclusively to microtubule, cilium, and cell projection assembly and organization (Table S4). Therefore, kinocilia maintenance appears to be a key difference between utricle hair cells that feature prominent kinocilia and basilar papilla hair cells, which do not possess kinocilia in their mature state.^23^^,^^24^

### Basilar papilla and utricle hair cell markers

**Figure 3:**
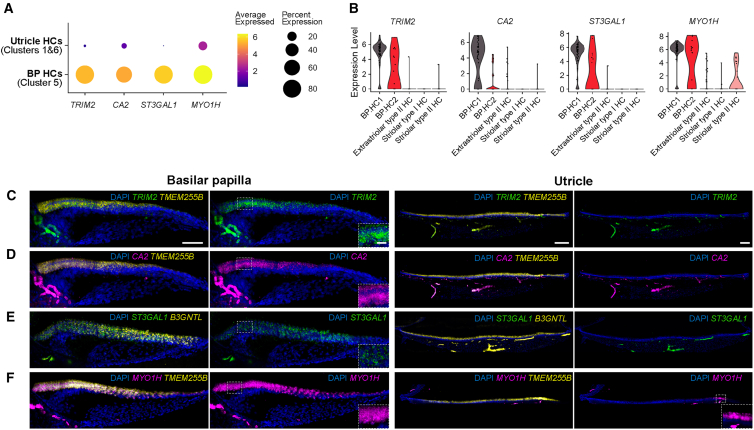
Markers for basilar papilla hair cells (A) Dot plot visualization of enriched genes in basilar papilla hair cells. The expression of *TRIM2*, *CA2*, *ST3GAL1*, and *MYO1H* in basilar papilla hair cells (BP HCs: cluster 5) and utricle hair cells (Utricle HCs: clusters 1 and 6) are shown. Dot size represents the percentage of cells expressing the gene, and the dot color represents average gene expression levels. (B) Violin plots show the expression levels of *TRIM2*, *CA2*, *ST3GAL1*, and *MYO1H* in individual hair cell subtypes. (C–F) *In situ* hybridization chain reaction (HCR) demonstrating high mRNA expression of *TRIM2* (C), *CA2* (D), *ST3GAL1* (E), and *MYO1H* (F) in basilar papilla hair cells, compared to the utricle hair cells. Inset images in the second row show magnified views of tall hair cells from the dotted boxed region of the basilar papilla. The inset image in the fourth row of panel (F) shows the magnified view of the hair cells in the striolar region of the utricle. Pan-hair cell markers *TMEM255B* or *B3GNTL* were detected to indicate the position of hair cells. Scale bar: 50 μm (basilar papilla), 10 μm (basilar papilla: magnified), 100 μm (utricle), and 10 μm (utricle: magnified).

We utilized hybridization chain reaction (HCR)^25^ to validate several markers that were found to be differentially expressed in auditory and vestibular hair cells *in situ*. *TMEM255B* mRNA expression was used as a distinct marker of hair cells because this gene is consistently and strongly expressed in all avian hair cell subtypes.^7^^,^^16^^,^^20^ Differential gene expression analysis indicated that *TRIM2*, *CA2*, *ST3GAL1*, and *MYO1H* were upregulated in basilar papilla hair cells compared to utricle hair cells (Figure 3A). HCR confirmed that mRNAs for these genes were specifically expressed in basilar papilla hair cells but absent in utricle hair cells (Figures 3C–3E), except for *MYO1H*, which was detectable in striolar hair cells of the utricle (Figure 3F). This was reflected in the single-cell dataset when we plotted *MYO1H* expression in all hair cell clusters. The results showed high expression in both BP hair cell clusters and relatively lower, yet specific, expression in the striolar type II HC cluster (Figure 3B).

**Figure 4:**
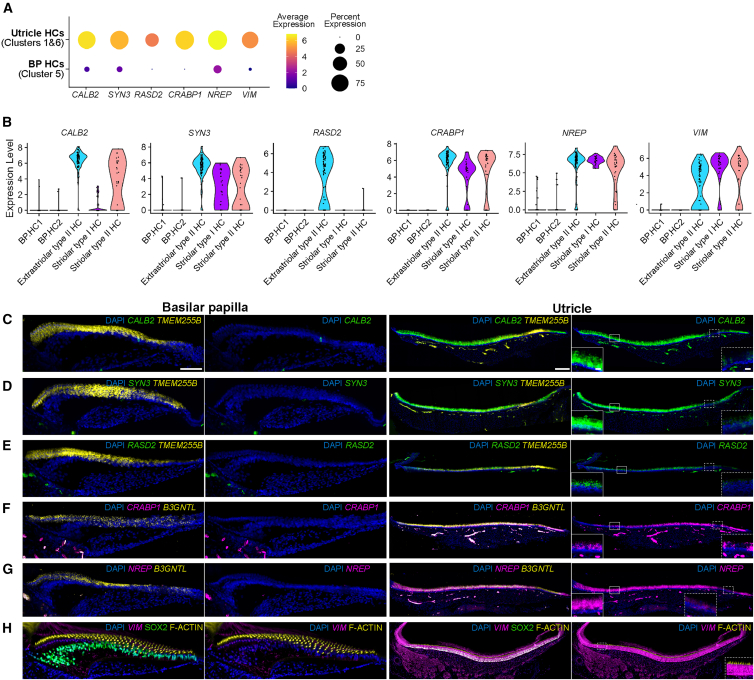
Markers for utricle hair cells (A) Dot plot visualization of enriched genes in utricle hair cells. The expression of *CALB2*, *SYN3*, *RASD2*, *CRABP1*, *NREP*, and *VIM* in the utricle and basilar papilla hair cells are shown. Dot size represents the percentage of cells expressing the gene, and the dot color average gene expression. (B) Violin plots show the expression levels of *CALB2*, *SYN3*, *RASD2*, *CRABP1*, *NREP*, and *VIM* in individual hair cell subtypes. (C–H) *In situ* hybridization chain reaction (HCR) demonstrating high mRNA expression of *CALB2* (C), *SYN3* (D), *RASD2* (E), *CRABP1* (F), *NREP* (G), and *VIM* (H) in utricle hair cells, compared to basilar papilla hair cells. Inset images in the fourth column, marked by solid and dotted boxes, show magnified views of the extrastriolar and striolar regions of the utricle, respectively. Pan-hair cell markers *TMEM255B* (C, D, E) or *B3GNTL* (F, G) were detected to indicate the position of hair cells. Hair bundles are labeled with F-actin (H). Scale bar: 50 μm (basilar papilla), 100 μm (utricle), and 10 μm (utricle: magnified).

Using the same strategy, we identified *CALB2*, *SYN3*, *RASD2*, *CRABP1*, *NREP*, and *VIM* as distinct markers for utricle hair cells and predicted their absence in the basilar papilla (Figures 4A and 4B). *In situ* HCR confirmed that these genes were exclusively expressed in utricle hair cells, not in basilar papilla hair cells (Figures 4C–4H). *CALB2* and *SYN3* were expressed at higher levels in extrastriolar hair cells than in striolar hair cells, consistent with the pattern observed in the single-cell dataset (Figure 4B). *RASD2* was identified as a specific marker for extrastriolar type II hair cells (Figures 4B and 4E). *CRABP1* expression was slightly higher in extrastriolar hair cells (Figures 4B and 4F). In contrast, *NREP* mRNA, which was computationally predicted at equally high levels in extrastriolar and striolar hair cells, was distinctively detected *in situ* at higher levels in extrastriolar hair cells compared to the striolar hair cells (Figures 4B and 4G). *VIM* was expressed in all hair cells throughout the utricle (Figures 4B and 4H). None of the six genes that we validated with HCR were detectable in basilar papilla hair cells.

### Nascent regenerated basilar papilla hair cells exhibit a unique transcriptomic profile that resembles that of utricle hair cells rather than basilar papilla hair cells

Basilar papilla hair cells can be ablated by infusing the ototoxic aminoglycoside sisomicin through the lateral semicircular canal.^26^ The ensuing hair cell demise triggers a coordinated regenerative response in supporting cells, leading to the formation of new hair cells, which are first detectable 96 h after the ototoxic insult.^16^^,^^27^^,^^28^ Note that the auditory brainstem response (ABR) is not detectable at this early stage of nascent hair cell emergence. Detectable hearing threshold recovery begins after 9 days post-sisomicin treatment (PST), with complete functional recovery occurring around 5 weeks PST.^28^ We integrated the single-cell data from these nascent basilar papilla hair cells at 96 h PST^27^ into our analysis to explore their transcriptomic similarity with the basilar papilla and utricle hair cells.

**Figure 5:**
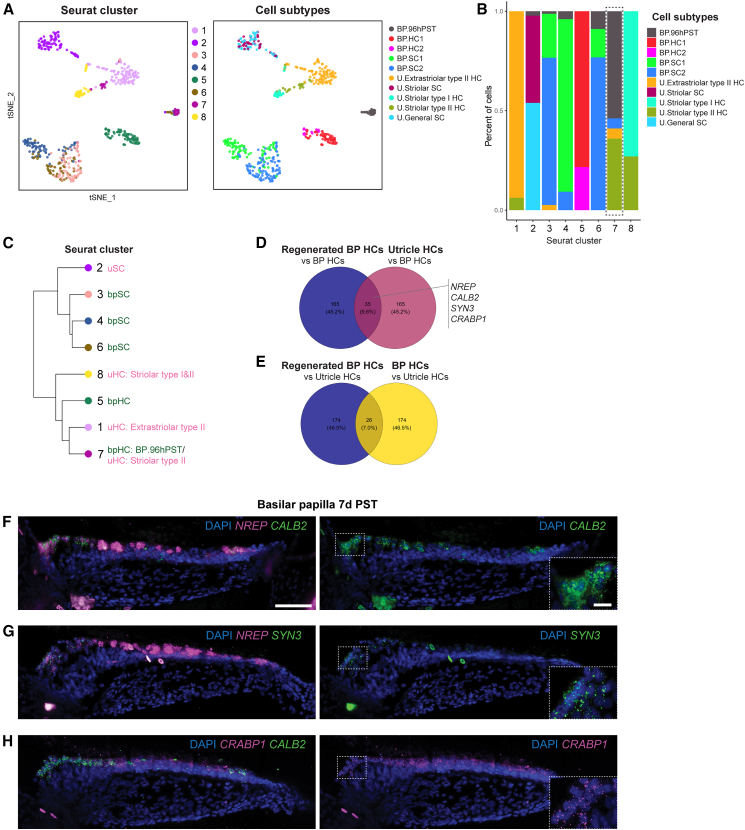
Upregulation of utricle hair cell markers in regenerated basilar papilla hair cells (A) t-SNE plots of the merged datasets of hair cells and supporting cells of the basilar papilla (BP), utricle in their homeostatic states, and nascent BP hair cells at 96 h post sisomicin treatment (PST), merged and re-clustered. The left panel shows the clustering predicted by Seurat, and the right panel shows the cell type annotation based on previously published CellTrails State. (B) Bar plot shows the proportion of cell subtypes in individual clusters. The Dotted box highlights cluster 7, where nascent basilar papilla hair cells and striolar type II hair cells of the utricle are grouped together. (C) Dendrogram shows the relationship of the clusters predicted by Seurat. Clusters 7 and 1 diverge from the same branch, indicating a close transcriptomic profile to each other. Primary cell subtypes in individual clusters are labeled. (D and E) The Venn diagram shows an overlap of 35 genes between the top 200 upregulated genes in regenerated basilar papilla hair cells PST compared to homeostatic basilar papilla hair cells, and the top 200 enriched genes in utricle hair cells compared to basilar papilla hair cells. This indicates that these 35 genes are utricle marker genes that are upregulated in regenerated basilar papilla hair cells but not expressed in homeostatic BP hair cells. *NREP*, *CALB2*, *SYN3*, and *CRABP1* were listed in the 35 overlapping genes (D). Similarly, 26 basilar papilla hair cell marker genes were upregulated in regenerated basilar papilla hair cells PST (E). (F–H) *In situ* hybridization chain reaction (HCR) demonstrates the expression of *NREP*, *CALB2*, *SYN3*, and *CRABP1* in regenerated basilar papilla hair cells 7 days PST. Dotted squares magnify regenerated basilar papilla hair cells. Scale bar: 50 μm (left) and 10 μm (right).

Clustering analysis of the combined data identified eight clusters (clusters 1–8) (Figure 5A; Table S5). Nascent basilar papilla hair cells (BP.96hPST) were primarily located in cluster 7, which also includes striolar type II utricle hair cells (Figure 5B). When we illustrated the cluster hierarchy in a dendrogram, we noticed that clusters 7 and 1 are most closely related (Figure 5C). Together, clusters 7 and 1 account for the majority of type II utricle hair cells, suggesting that nascent basilar papilla hair cells exhibit a distinct transcriptomic profile similar to that of utricle hair cells, particularly the type II subtype. Conversely, this also implies that mature utricle type II hair cells express genes that are transiently expressed in nascent basilar papilla hair cells.

We further elaborated on this finding by comparing the top 200 differentially expressed genes among newly regenerated nascent, mature basilar papilla, and utricle hair cells (Table S6). Thirty-five genes were differentially expressed by nascent hair cells and utricle hair cells but were not detected in mature basilar papilla hair cells (Figure 5D; Table S6). Among these were validated utricle hair cell marker genes, including *NREP*, *CALB2*, *SYN3*, and *CRABP1* (Figures 4C–4F). Conversely, we also identified 26 genes that are expressed by nascent hair cells and mature basilar papilla hair cells but not in utricle hair cells (Figure 5E; Table S6). Using HCR, we validated the upregulation of *NREP*, *CALB2*, *SYN3*, and *CRABP1* in nascent hair cells in the basilar papilla (Figures 5F–5H).

We then integrated another group of nascent hair cells, which were previously identified in the homeostatic utricle as new nascent hair cells arising due to the daily turnover of hair cells (U.S6 in Figure 1B).^20^ Clustering analysis revealed that the majority of nascent basilar papilla and utricle hair cells grouped together in a single cluster (cluster 5), along with a small portion of mature striolar type II hair cells of the utricle (Figures S2A and S2B; Table S7). Notably, a subset of nascent basilar papilla hair cells clustered together with basilar papilla supporting cells (clusters 1 and 2). This relationship is consistent with their lineage, as basilar papilla hair cells originate from the supporting cells. The nascent cells in clusters 1 and 2 likely represent an earlier stage of this regenerative process when they still retain some transcriptional features of their supporting cell origin. To verify the similarity between nascent basilar papilla hair cells (BP.96hPST) and nascent utricle hair cells (U.differentiating HC), differentially expressed gene analysis was performed between these two groups within cluster 5. As expected, only a small number of differentially expressed genes were identified in each group, including their respective mature counterparts’ marker genes, such as *VIM* (mature utricle hair cell marker; Figure 2B; Table S8), and *NR2F2*, *GATA3*, and *S100A1* (mature basilar papilla hair cell markers; Figure 2B) (Figure S2C). Hierarchical clustering indicated that the cluster with nascent hair cells is closest to extrastriolar type II hair cells (Figure S2D). These results suggest that the newly regenerated nascent hair cells from the basilar papilla and the nascent utricle turnover hair cells share a similar transcriptomic profile during early differentiation.

### Regenerated hair cells in the utricle express utricle type II hair cell marker genes

**Figure 6:**
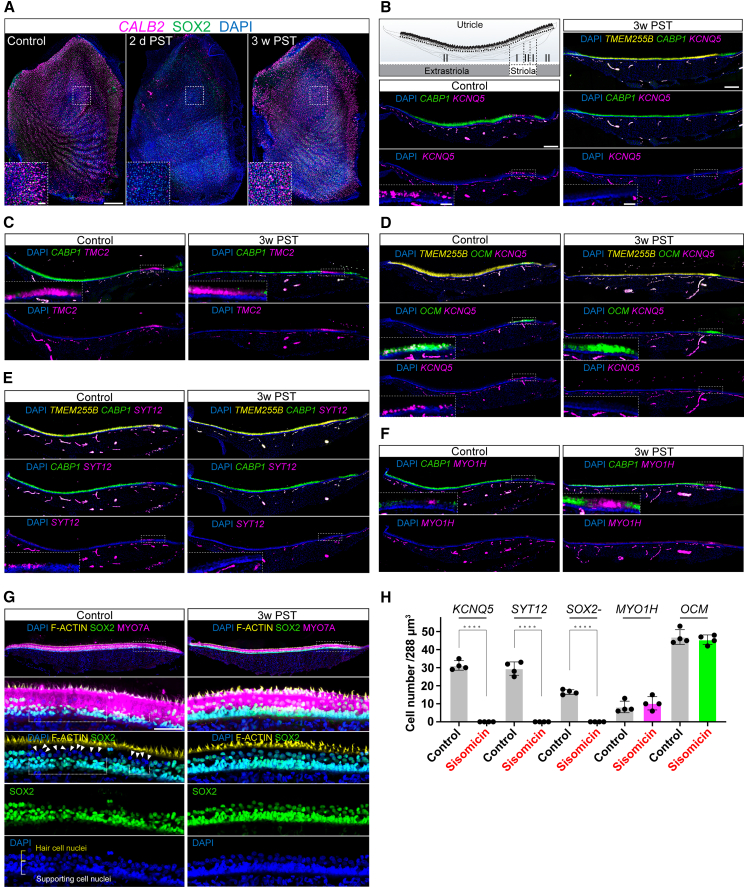
Regenerated utricle hair cells no longer show type I properties by three weeks after damage (A) *In situ* HCR of the whole-mount utricles presenting the loss of *CALB2*-expressing hair cells at two days post-sisomicin treatment (2d PST), followed by their reappearance at three weeks post-treatment (3w PST) throughout the utricle. Scale bar: 200 μm and 30 μm (magnified). (B) A schematic illustrating the transverse section of the chicken utricle and the anatomical location of hair cell subtypes (top left). *In situ* hybridization chain reaction (HCR) demonstrating the mRNA expression of *TMEM255B* across the utricle and *CABP1* in the extrastriola of the utricle at 3 weeks PST. *KCNQ5* expression was absent at 3 weeks PST. Scale bar: 100 μm and 20 μm (magnified). (C–F) *In situ* HCR demonstrating the mRNA expression of *TMC2* (C) and *OCM* (D) in striola at 3 weeks PST. The expression of *KCNQ5* (D) and *SYT12* (E) were absent, while the increased expression of *MYO1H* (F) was observed in striola at 3 weeks PST. (G) Immunostaining imaging showing that all hair cells in the striola express SOX2 at 3 weeks PST, with no SOX2-negative hair cells observed. The second row images show enlarged views of the areas outlined with dotted squares in the first row images. Dotted areas in the control image indicate striolar regions where type I HCs reside. Scale bar: 25 μm. Arrowheads in the control image of the third row indicate SOX2-negative hair cell nuclei, whereas all hair cells in the image at 3 weeks PST are positive for SOX2. The fourth and fifth row images show the individual channels for SOX2 and DAPI, respectively. (H) Quantification of the number of hair cells per 288 μm^3^ (120 × 40 × 60 μm area) in striola expressing *KCNQ5*, *SYT12*, *MYO1H*, and *OCM*, as well as hair cells lacking SOX2 expression, in the utricles at 3 weeks PST and in the contralateral control. Means ± SDs are shown. *n* = 4. The statistical analysis was performed using one-way ANOVA. ^∗∗∗∗^*p* < 0.0001.

Since newly regenerated basilar papilla hair cells and utricle hair cells generated during natural turnover display a similar transcriptomic profile that recapitulates the expression of some markers for utricle type II hair cells, we were curious to investigate the gene expression pattern of utricle hair cells that regenerate after sisomicin-induced loss. In these experiments, we relied on the HCR-based detection of marker genes in utricles at three weeks post-treatment. Two days after sisomicin treatment, hair cells were lost throughout the utricle, as indicated by the absence of *CALB2*-expressing cells (Figure 6A).

By three weeks post-treatment, we confirmed the emergence of new hair cells by detecting *TMEM255B*-positive hair cells and the reappearance of *CALB2*-positive cells (Figures 6A–6E). Although the three-week timepoint does not reflect the very early stage of newly regenerated basilar papilla hair cells and turnover-generated utricle hair cells, it provided an opportunity to test whether three-week-old regenerated utricle hair cells assume a mature gene expression phenotype. At three weeks after hair cell ablation, the expression of *CABP1*, an extrastriolar type II hair cell marker,^20^ labeled the regenerated hair cells in the extrastriola, similar to the control utricle from the contralateral undamaged inner ear (Figure 6B). The striolar hair cell markers *TMC2* and *OCM*^20^ were expressed in the regenerated striolar hair cells (Figures 6C and 6D). However, the expression of *KCNQ5*, a striolar type I hair cell marker,^20^ was not detected in regenerated striolar hair cells (Figures 6B and 6D). Moreover, another type I striolar hair cell marker, *SYT12*,^20^ was also absent from regenerated striolar hair cells (Figure 6E).

These results indicate that while regenerated hair cells in the striola acquired some location-specific properties, they failed to develop into type I hair cells. In addition, we observed an enlargement of the *MYO1H* expression domain, indicative of striolar type II hair cells,^20^ in the regenerated striola compared to the control (Figure 6F). The absence of SOX2 expression is a hallmark of mature type I hair cells. At three weeks post-ablation, we did not detect any SOX2-negative hair cells; instead, SOX2 expression was observed in all the striolar hair cells (Figure 6G). The quantification of the SOX2-negative hair cells in the control versus sisomicin-treated samples confirmed these observations, supporting the notion that regenerated hair cells lack type I properties (Figure 6H). Taken together, our analysis suggests that regenerated extrastriolar hair cells in the utricle at three weeks post hair cell ablation display a type II profile, which is expected because it reflects the natural cell type distribution in birds. In the striola, in contrast, we did not detect gene expression patterns that coincide with type I hair cells. Instead, we observe a distinct group of type II hair cells that express striolar markers such as *TMC2*, in addition to a subset of hair cells that express *MYO1H*.

## Discussion

By leveraging published single-cell RNA sequencing datasets of the chicken basilar papilla and utricle for comparative analyses, we have characterized the differences in gene expression profiles between hair cells and supporting cells in these two organs. We found substantial differences in gene expression among hair cell subtypes. Supporting cells also displayed differences, although basilar papilla supporting cells showed less variation. This may reflect specific functional characteristics and could serve as a basis for future investigations. For the purpose of this study, we provide these differences (Figure S1; Table S3), but we focused our in-depth analysis and validation on hair cells. Furthermore, we included two groups of nascent hair cells in our study: regenerated nascent basilar papilla hair cells and hair cells emerging through natural turnover in the utricle, providing a comparison of these juvenile hair cells after regeneration and natural homeostasis.

Despite the shared role of mechanotransduction in auditory and vestibular hair cells, our analysis identified substantial gene expression differences between these hair cell subgroups, which would be reasonable given their specialized function. Enriched GO terms in utricle hair cells indicated active maintenance or formation of cilia, which likely refers to the kinocilium, a microtubule-based cilium critical for hair cell development, orientation, and function of vestibular hair cells.^29^ In contrast, basilar papilla hair cells showed the enrichment of a single GO term related to cell surface modification via sialylation. Since the cellular glycome influences various processes in hearing^30^ and other systems, including cell-cell recognition and signal transduction,^31^ such processes might be post-translationally fine-tuned through glycosylation, particularly through sialylation in basilar papilla hair cells. It is difficult to speculate further without knowing more details. One context could involve different interactions with overlying structures: the tectorial membrane in the basilar papilla and the gelatinous otolithic membrane overlying utricle hair cells.

Our results consistently indicated that nascent hair cells have a transcriptomic profile closer to that of utricle type II hair cells than to the other hair cell types. Particularly, newly regenerated nascent basilar papilla hair cells exhibited similarity to utricle type II hair cells, which was further supported by the upregulation of multiple utricle hair cell marker genes. Among these genes was cellular retinoic acid binding protein 1 (*CRABP1*), a key component of the retinoic acid signaling pathway,^32^ which promotes cell cycle progression in stem cells^33^ and regulates apex-to-base patterning in the embryonic cochlea and hair cell differentiation in mammals.^34^^,^^35^ Genes such as neuronal regeneration-related protein (*NREP*), which is expressed in neural tissues or muscles and promote the G1/S phase transition of the cell cycle.^36^ Our transcriptomic analyses also identified eye absent homolog 1 (*EYA1*), which cooperates with *Six1* and *Sox2* to activate *Atoh1* for the induction of cochlear hair cells,^37^^,^^38^ and transcription factors, such as *CREB5*, *BCL11A*, and *HMGB2*. It is reasonable to conclude that newly regenerated basilar papilla hair cells and nascent hair cells in the utricle undergo a transient but distinct phase that is marked by a specific gene expression profile. As the cells mature and grow hair bundles, as well as establish synaptic contacts, these changes are likely accompanied by changes in their gene expression profiles. It is interesting, however, that the transient nascent transcriptional profile is well-defined, and that cells from different organs (auditory and vestibular) arising in different contexts (regeneration and natural turnover) are clustered together.

In the utricle, we did not find evidence of a type I hair cell phenotype in regenerated new hair cells after three weeks. This finding is consistent with an earlier study using ^3^H-thymidine autoradiography and bromodeoxyuridine (BrdU) labeling in the chicken utricle, which demonstrated that the hair cells generated through natural turnover are exclusively type II hair cells characterized by bouton-like innervation.^39^ Another study in pigeons reported the emergence of type II hair cells in the regenerating vestibular sensory epithelium after aminoglycoside-induced damage, as confirmed by the type II electrophysiological properties of these cells.^40^ In mouse utricles, multiple lines of evidence have demonstrated that the supporting cells can give rise to type II hair cells, while type I hair cells are hardly replenished.^41^^,^^42^^,^^43^ In our study, we identified regenerated hair cells in the striola that could not be clearly classified as either type I or type II, but expressed striolar hair cell markers at three weeks post-aminoglycoside administration. A previous long-term study of regenerating chicken utricles showed an increase of type I hair cells between 20 and 60 days after aminoglycoside-induced hair cell injury, suggesting that some type II hair cells may convert into type I hair cells over time.^44^ Vestibular recovery has been reported to be a slow process, and vestibular dysfunction in birds persists after 3–4 weeks post hair cell ablation.^45^ It is possible that the undefined hair cells we observed may represent an early phase of this transition. Overall, together with the previous evidence, our results indicate that nascent hair cells in the chicken basilar papilla and utricle transiently display utricle type II gene expression properties. This, in turn, suggests that utricle type II hair cells may retain a more primitive and plastic state compared to type I hair cells, potentially representing a less evolved state of this sensory cell type.

Our study presents a comparative transcriptomic atlas of chicken basilar papilla and utricle hair cells. We provide baseline properties of avian inner ear hair cells in homeostasis and during regeneration, which can serve as a foundation for classifying hair cell subtypes in more elaborate cross-species comparisons.^46^

### Limitations of the study

Transcriptomic analysis performed in this study identified distinct gene expression patterns in cochlear and vestibular hair cells in chickens. We focused on the basilar papilla and the utricle, which are the most extensively studied inner ear organs, but we acknowledge that the study is limited by the lack of single-cell data and the absence of detailed regenerative studies on the vestibular cristae, saccule, and lagena. We confirmed the expression of the identified markers in their respective tissues. Although the functional roles of these genes were predicted through GO term analysis, further investigations, such as overexpressing or depleting the genes to assess their impact on hearing outcomes, are required. However, genetic manipulation tools for avian species are limited. While using viral vectors is a potential approach, their efficacy and tropism for specific inner ear cell types remain to be fully characterized. Our study also identified gene expression differences among the supporting cell subgroups of the chicken cochlea and vestibule. However, cochlear supporting cells exhibited a less distinct profile compared to the utricular supporting cells. This suggests that in-depth analyses, such as chromatin accessibility, together with the comprehensive examination and validation of the current data, are necessary to provide in-depth analyses of supporting cell subtypes. Our data suggested a potential conversion from type II to type I hair cell subtypes following the emergence of nascent hair cells after aminoglycoside-induced damage. To verify this hypothesis, a longer-term examination of type I hair cell re-emergence, including cytomorphological and functional analyses in addition to gene expression profiling, would be necessary.

## Resource availability

### Lead contact

Further information and requests may be directed to the lead contact, Marie Kubota (kubomari@stanford.edu).

### Materials availability

The study did not generate new unique reagents.

### Data and code availability

• Data: No new data were generated for this study. The Single-cell RNA-seq data presented have been published in the referenced articles,^7^^,^^20^^,^^27^ where the data deposition details are provided; see key resources table.
• Code: No original code was developed.
• Other items: N/A.

## Acknowledgments

We thank the Stanford Otolaryngology Imaging Core for their expert assistance with imaging. M.P.S. acknowledges support from the Soda Toyoji SPIO Scholarship 2022 (no. SS22002), 10.13039/501100001691JSPS KAKENHI Grant Number JP25K20152, and 10.13039/100012043Kindai University Research Enhancement Grant (SR01). M.K. received support from the 10.13039/100000002National Institutes of Health (DC020271), 10.13039/501100001691Japan Society for the Promotion of Science (Overseas Research Fellowships), the 10.13039/100008732Uehara Memorial Foundation (Overseas Research Fellowships), and the 10.13039/100028926Soda Toyoji Memorial Foundation. This work was supported by National Institutes of Health Grant DC019619 to S.H.

## Author contributions

Conceptualization, M.P.S, I.V.H, S.H., and M.K.; methodology, M.P.S, I.V.H, and M.K.; formal analysis, M.P.S, I.V.H, and M.K.; investigation, M.P.S, I.V.H, S.H., and M.K.; visualization, M.P.S, I.V.H, and M.K.; funding acquisition, M.P.S, S.H., and M.K.; project administration, S.H. and M.K.; supervision, S.H. and M.K.; writing – original draft, M.P.S, I.V.H, S.H., and M.K.; writing – review and editing, M.P.S, I.V.H, S.H., and M.K. All authors have read and approved the final version of the article.

## Declaration of interests

The authors have no conflicts or conflicting interests to declare.

## Declaration of generative AI and AI-assisted technologies in the writing process

During the preparation of this work, the authors used Grammarly to check for typos and grammatical errors and to improve readability. After using this tool, the authors reviewed and edited the content as needed and take full responsibility for the content of the publication.

## STAR★Methods

### Key resources table

**Table:** 

REAGENT or RESOURCE	SOURCE	IDENTIFIER
**Antibodies**		

Rabbit polyclonal anti Myosin-7a (1:500)	Proteus Biosciences	Cat# 25-6790; RRID: AB_10015251
Mouse monoclonal anti-Sox2-488 (1:200)	Santa Cruz Biotechnology	Cat# sc-365823; RRID: AB_10842165
Donkey anti-Rabbit IgG, Alexa Fluor 546 (1:500)	Thermo Fisher Scientific	Cat# A10040; RRID: AB_2534016
Donkey anti-Mouse IgG, Alexa Fluor 488 (1:500)	Thermo Fisher Scientific	Cat# A-21206; RRID: AB_141607
Alexa Fluor 647 Phalloidin (1:50)	Invitrogen	Cat# A22287; RRID: AB_2620155
Vimentin Polyclonal antibody	proteintech	Cat# 10366-1-AP
Rabbit polyclonal anti-calretinin (1:1000)	Swant	Cat number 7697; RRID: AB_305702
DAPI (1:1000)		

**Oligonucleotides**		

CALB2 HCR DNA probe	NM_205316.2	Lot# PRJ052
TMEM255B HCR DNA probe	XM_040657646.2	Lot# RTB850
NREP HCR DNA probe	NM_001397042.1	PRJ059
SYN3 HCR DNA probe	XM_040660768	RTD099
CRABP1 HCR DNA probe	NM_001030539.2	RTH707
RASD2 HCR DNA probe	XM_040660760.2	PRD382
CABP1 HCR DNA probe	XM_004934386	Lot# PRD383
KCNQ5 HCR DNA probe	XM_015284764.4	PRD381
TMC2 HCR DNA probe	NM_001039324.2	PRD378
OCM HCR DNA probe	NM_204422.1	PRJ051
SYT12 HCR DNA probe	NM_001199512.2	PRD380
MYO1H HCR DNA probe	XM_040684691.2	RTH706
TRIM2 HCR DNA probe	NM_001257314.2	RTH703
CA2 HCR DNA probe	NM_205317.2	RTH704
ST3GAL1 HCR DNA probe	NM_205217.1	RTH705
B3GNTK HCR DNA probe	NM_001277651.1	Lot # RTA482

**Chemicals, peptides, and recombinant proteins**		

Paraformaldehyde	EMS	Cat# 15710
Low Melt Agarose	BioRad	Cat# 1613112
Mounting Media (Citifluor CFM-3)	EMS	Cat# 17979-30
Isoflurane, USP	VetOne	Cat# 502017
Carprofen	Millipore Sigma	Cat# SML1713
Sisomicin	MilliporeSigma	Cat# 1612801
Surgical superglue	VetClose, Henry Schein Animal Health	Cat# BS031477
20X Sodium chloride citrate (SSC)	Thermo Fisher Scientific	Cat# AM9770
Proteinase K, molecular biology grade (800 units/mL)	New England Biolabs	Cat# P8107S
HCR probe hybridization buffer	Molecular Instruments	N/A
HCR probe wash buffer	Molecular Instruments	N/A
HCR amplification buffer	Molecular Instruments	N/A

**Deposited data**		

Chicken basilar papilla sensory epithelial cells	Janesick et al.^7^	gEAR: https://umgear.org/p?l=b073d741
Chicken utricle sensory epithelial cells, nascent chicken utricle hair cells during natural turnover	Scheibinger et al.^20^	GEO: GSE212831 gEAR: https://umgear.org/p?l=GgUtricleP7 Mendeley Data: https://data.mendeley.com/datasets/zypn85x7y7
Nascent chicken basilar papilla hair cells post-sisomicin treatment	Janesick et al.^27^	gEAR: https://umgear.org/p?l=5d177e1c&g Zenodo: https://doi.org/10.5281/zenodo.5504624

**Experimental models: Organisms/strains**		

Fertilized chicken eggs, SPF, Premium	Charles River	10100326

**Software and algorithms**		

Fiji/ImageJ	Fiji	RRID:SCR_002285https://fiji.sc
GraphPad Prism	Prism_Version 8.4.1	https://www.graphpad.com/scientific-software/prism/
Black Zen	Zeiss	https://www.zeiss.com/microscopy/en_us/products/microscope-software/zen.html; RRID:SCR_018163
R	R Project for Statistical Computing	RRID:SCR_001905 Version 4.2.0
RStudio	https://www.rstudio.com/	2024.09.01 Build 394
Seurat	https://cran.r-project.org/web/packages/Seurat/	RRID:SCR_016341 Version 5.1.0
scCustomize	https://samuel-marsh.github.io/scCustomize/	RRID:SCR_024675 Version 3.0.1
clusterProfiler	http://bioconductor.org/packages/release/bioc/html/clusterProfiler.html	RRID:SCR_016884 Version 4.12.6
CellTrails	https://www.bioconductor.org/packages/devel/bioc/html/CellTrails.html	Version 1.22.0
SeuratWrappers	https://github.com/satijalab/seurat-wrappers	RRID:SCR_022555 Version 0.3.5

### Experimental model and study participant details

All animal care and procedures complied with the Guide for the Care and Use of Laboratory Animals published by the National Institutes of Health. Chicken hatching, housing, and animal procedures were approved by the Stanford University Institutional Animal Care and Use Committee and performed as described.^28^ We took all necessary steps to minimize animal suffering, including the use of anesthesia and analgesia during surgical procedures, and careful monitoring of animal health and welfare. Both male and female animals were included in all analyses.

### Method details

#### Animals

Fertilized chicken eggs (Charles River, Wilmington, USA) were placed in a humidified incubator set at 38 °C, with automatic rocking. On day 19 of incubation, eggs were transferred to a Brinsea Octagon incubator for hatching. After the chicks were dry, they were moved to a cage equipped with an infrared heat lamp, a digital thermometer, chick starter feed, and water. The chickens were raised under a 12-h artificial day-night cycle in a low-ambient sound environment.

#### Surgical procedures

Seven-day-old chickens were anesthetized with 3% isoflurane and 2 L/min oxygen delivered via a nose cone while positioned laterally on a heating pad. Anesthesia was maintained by administering 2% isoflurane and 2 L/min oxygen. Feather removal in the postauricular area was accomplished using Nair, followed by three alternating applications of diluted betadine and 70% isopropanol. A small incision was made to expose the surface of the temporal bone. The lateral semicircular canal was identified above the subarcuate artery and punctured with a 30 ^1^/_2_-gauge needle, leading to a slight discharge of perilymph. Infusion of 75 μg/μL sisomicin in a total volume of 2 μL was carried out through the lateral semicircular canal using a Micropump (UMP3Micro 4, World Precision Instruments) and a 35-gauge blunt Nanofil needle (World Precision Instruments) at a rate of 8 nL/s. The puncture wound was sealed with bone wax (Ethicon), and the skin wound was closed using surgical superglue. Just before the surgery concluded, carprofen at 1 mg/kg was administered subcutaneously near the breast under the wing. Each chicken was labeled with colored leg bands (Chicken Hill) for identification and closely monitored for 15 minutes before being returned to the cage.

#### Dissection of chicken temporal bones

The chickens were euthanized with CO_2_ inhalation, decapitated, and the heads were bisected. The excised temporal bone, including the basilar papilla and utricle, was placed into 4% paraformaldehyde in phosphate-buffered saline (PBS) for fixation and stored at 4°C overnight. The samples were rinsed with PBS three times and then stored in PBS at 4°C until further processing. The temporal bone on the right side served as an age-matched control.

#### Vibratome sectioning

The basilar papilla or utricles with otoconia were extracted from the temporal bone and embedded in a disposable container filled with a 4% low-melt agarose solution in PBS. Note that the 4% agarose was maintained in a water bath set at 55°C until use. Each sample was arranged upright, side by side in the container and then stored at 4°C until the agarose solidified. After trimming an agarose block, transverse sectioning was performed using a Leica VT 1200 vibratome, with parameters set to 60-70 mm thickness, 1 mm amplitude, and a cutting speed of 0.7 mm/s. The sample slices were punched out using a 1.5 mm biopsy punch on a Sylgard 184 silicone plate.

#### Immunohistochemistry

The transverse sections of the basilar papilla or utricle were permeabilized with PBS containing 0.5% Triton (PBST) for 30 minutes at room temperature. The samples were immersed in a blocking buffer containing 1% BSA for 30 minutes, followed by an overnight incubation with primary antibodies (MYO7A (1:500, Proteus Biosciences), VIM (1:200, Proteintech), and SOX2 (1:200, Santa Cruz Biotechnology)) at 4°C with gentle rotation. After washing three times with 0.2% PBST, the samples were incubated with secondary antibodies and DAPI for two hours at room temperature. Following three washes with 0.2% PBST, the samples were mounted on glass slides using Mounting Media (Citifluor CFM-3), employing a 0.12 mm spacer between the slide and coverslip.

#### *In situ* hybridization chain reaction (HCR)

Transverse vibratome sections of the basilar papilla or utricle were prepared in the same manner as described above, under an RNase-free environment. The samples were dehydrated in 100% methanol on ice and stored at -20°C overnight. They were transferred to a glass slide and surrounded by a water-repellent barrier created with a PAP pen. The samples adhered tightly to the glass slides and were rehydrated using a series of graded methanol in PBS with 0.1% Tween for five minutes on ice: 75%, 50%, and 25% methanol, followed by PBS with 0.1% Tween. The rehydrated samples were treated with a 20 μg/mL proteinase K solution for 10 minutes at room temperature, then postfixed with 4% PFA for five minutes. After two washes on ice with PBS containing 0.1% Tween, 5X sodium saline citrate with 0.1% Tween 20 (SSCT) was applied for five minutes. Each mRNA HCR probe, purchased from Molecular Instruments, was prepared by diluting 1 μL of stock solution in 200 μL of 30% probe hybridization buffer. The samples were first incubated in 30% probe hybridization buffer for 30 minutes at 37°C, then immersed in the buffer containing mRNA probes at 37°C overnight. The following mRNA probes were used: *TRIM2* (Alexa Fluor® 488), *ST3GAL1* (Alexa Fluor® 488), *MYO1H* (Alexa Fluor® 546), *CA2* (Alexa Fluor® 546), *TMEM255B* (Alexa Fluor® 647), *B3GNTL* (Alexa Fluor® 647), *CALB2* (Alexa Fluor® 488), *SYN3* (Alexa Fluor® 488), *RASD2* (Alexa Fluor® 488), *NREP* (Alexa Fluor® 546), *CRABP1* (Alexa Fluor® 546), *OCM* (Alexa Fluor® 488), *SYT12* (Alexa Fluor® 546), *MYO1H* (Alexa Fluor® 546), and *TMC2* (Alexa Fluor® 546). The samples were washed with probe wash buffer four times to remove excess probes at 37°C, then incubated in 5X SSCT for 10 minutes. After preamplification with amplification buffer, the samples were treated with preheated and cooled H1 and H2 hairpins in amplification buffer, followed by incubation at room temperature overnight. After two washes with 5X SSCT, the samples were incubated with DAPI for one hour, and then mounted with Mounting Media (Citifluor CFM-3), followed by placing a coverslip on top of a 0.12 mm spacer.

#### Confocal microscopy

The sections were imaged with a LSM700 confocal laser scanning microscope (ZEISS) and processed with Fiji software. The representative images are presented from more than three replicates.

#### scRNA-seq and data processing

Single-cell isolation and sequencing were carried out as previously described (Janesick et al., 2022; Scheibinger et al., 2022). In brief, individual cells were sorted into 96-well plates and processed using the Smart-seq2 protocol to generate single-cell libraries. Paired-end sequencing (2 × 150 bp) was performed, and the resulting FASTQ reads were aligned to the NCBI Gallus gallus v6.0 reference genome using the STAR aligner. Gene-level read counts were quantified with RSEM.

### Quantification and statistical analysis

The basilar papilla and utricle single-cell datasets were subset based on previously published annotations, and only the raw count data were imported and merged for a new analysis using Seurat v5.0. The merged gene expression count matrices were processed using the SCTransform workflow in Seurat. Dimensionality reduction was performed using PCA, and the first 10 principal components were used to create a k-nearest neighbor graph based on the Euclidean distance metric, along with a shared nearest neighbor graph of the cells in the PC space. The cells were clustered using the Louvain algorithm with multiple values for the resolution parameter, and a value of 0.8 was chosen, which yielded the most biologically meaningful clustering. The clustering results were visualized using UMAP and tSNE algorithms. We used the plotting functions in the R package scCustomize to create plots.^47^ Differential gene expression analysis was conducted to identify marker genes for all clusters using the Wilcoxon rank-sum test using the presto R package.^48^ GO term analysis was performed with the clusterProfiler R package.^49^

## Supplemental information

Document S1. Figures S1 and S2

Table S1. Marker genes for clusters 1–7 identified in a combined dataset of the basilar papilla and utricle hair cells and supporting cells, related to Figure 1

Table S2. Marker genes for basilar papilla and utricle hair cells, related to Figure 2 Differentially expressed genes in cluster 5 (basilar papilla hair cells) versus clusters 1 and 6 (utricle hair cells). Thresholds are set to avg_log2-FC >|2|.

Table S3. Marker genes for basilar papilla and utricle supporting cells, related to Figure S1 Differentially expressed genes in clusters 2, 4, and 7 (basilar papilla supporting cells) versus cluster 3 (utricle supporting cells). Thresholds are set to avg_log2-FC >|2|.

Table S4. GO terms showing biological processes enriched in genes of basilar papilla hair cells (cluster 5) compared to utricle hair cells (clusters 1 and 6), and of utricle hair cells (clusters 1 and 6) compared to basilar papilla hair cells (cluster 5), related to Figure 2 Adjusted p-value <0.05.

Table S5. Marker genes for clusters 1–8 identified in a combined dataset of the basilar papilla and utricle hair cells and supporting cells, in addition to regenerated basilar papilla hair cells at 96 h post sisomicin treatment, related to Figure 5

Table S6. List of genes included in Venn diagrams in Figure 5D, related to Figure 5 (List 1) Top 200 differentially expressed genes in regenerated basilar papilla hair cells compared to basilar papilla hair cells (logFC>|2| and adj_p_value = 1E-4). (List 2) Top 200 differentially expressed genes in utricle hair cells compared to basilar papilla hair cells. (List 3) The overlapping 35 genes in List 1 and List 2. (List 4) Top 200 differentially expressed genes in regenerated basilar papilla hair cells compared to utricle hair cells. (List 5) Top 200 differentially expressed genes in basilar papilla hair cells compared to utricle hair cells. (List 6) The overlapping 26 genes in List 1 and List 2.

Table S7. Marker genes for clusters 1–7 identified in a combined dataset of the basilar papilla and utricle hair cells and supporting cells, regenerated basilar papilla hair cells at 96 h post sisomicin treatment, and differentiating utricle hair cells, related to Figure S2

Table S8. Marker genes for regenerated basilar papilla and nascent utricle hair cells during natural turnover, related to Figure S2 Differentially expressed genes within cluster 5, comparing regenerated basilar papilla hair cells (BP.96hPST) versus nascent utricle hair cells (U.Differentiating HC).
