# Supplementary material for: Transcriptomic comparison of avian auditory and vestibular sensory epithelia
Source: iScience. 2025 Oct 15;28(11):113780. doi: 10.1016/j.isci.2025.113780 (PMC12616014; doi:10.1016/j.isci.2025.113780)
Supplement: Document S1. Figures S1 and S2 [file mmc1.pdf]

iScience, Volume *28*

Supplemental information

Transcriptomic comparison of avian auditory and vestibular sensory epithelia

Mitsuo Paul Sato, Ishwar Vithal Hosamani, Stefan Heller, and Marie Kubota

Figure S1 Differentially expressed genes in the basilar papilla and utricle supporting cells.

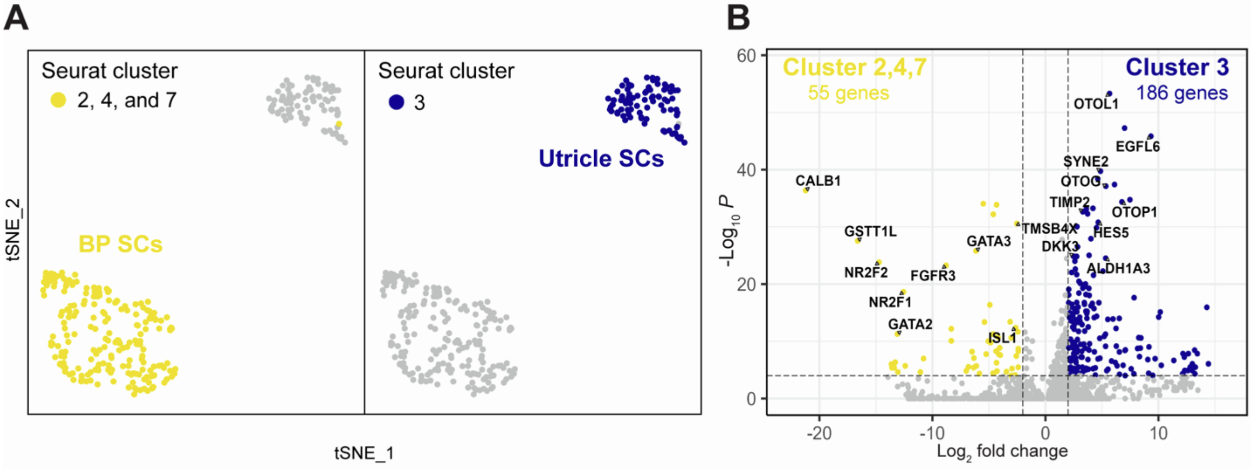

(A) tSNE plots highlighting basilar papilla supporting cells (cluster 5) and utricle hair cells (clusters 1 and 6).

(B) Volcano plot showing DE genes between basilar papilla supporting cells and utricle supporting cells. 55 and 186 DE genes were identified in basilar papilla and utricle supporting cells, respectively (Log_2 fold change >|2| and p_val_adj <1E-4).

Related to Figure 2.

Figure S2 **Regenerated basilar papilla hair cells, differentiating utricle hair cells, and type II hair cells of utricle have similar transcriptomic profiles.**

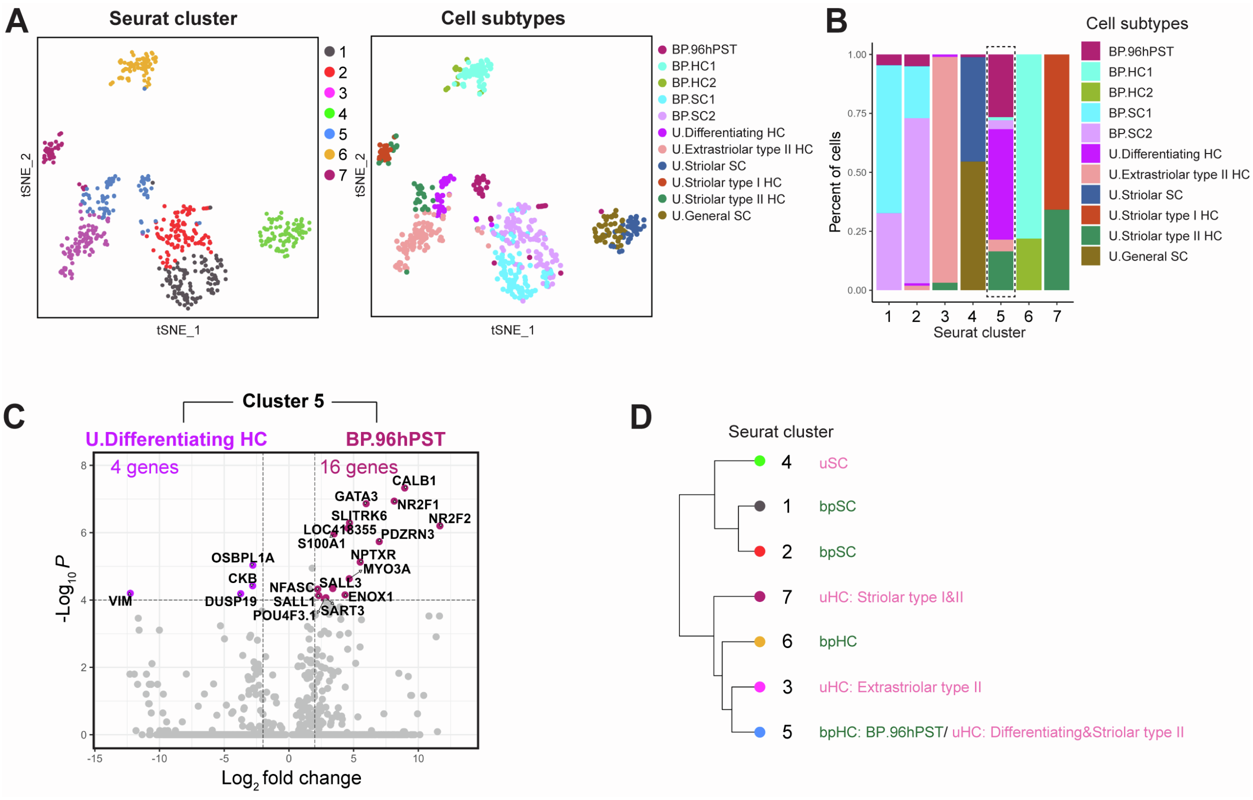

(A) UMAP, including hair cells and supporting cells of the basilar papilla (BP) and utricle in their homeostatic states, regenerated BP hair cells at 96 hours post sisomicin treatment (PST), and differentiating utricle hair cells during natural turnover, merged and re-clustered, visualized by Seurat clusters (left) and cell subtypes (right).

(B) Bar plot showing the proportion of cell subtypes in individual clusters. Dotted box highlights cluster 5 where nascent basilar papilla and utricle hair cells are grouped together.

(C) Volcano plot showing differentially expressed (DE) genes between regenerated basilar papilla hair cells (BP.96hPST) and nascent utricle hair cells (U.Differentiating HC). 16 and 4 DE genes were identified in regenerated basilar papilla and nascent utricle hair cells, respectively (Log_2 fold change >|2| and p_val_adj <1E-4).

(D) Dendrogram showing the proximity of the clusters. Clusters 5 and 3 diverge from the same branch, indicating a close transcriptomic profile to each other. Primary cell subtypes in individual clusters are labeled.

Related to Figure 5.
